# Beyond Coiling: A Comparative Analysis of Survey-Reported Preferences for Endovascular Cerebral Aneurysm Occlusion

**DOI:** 10.3390/clinpract16060112

**Published:** 2026-06-15

**Authors:** Sanjana R. Salwi, Thilan Tudor, Oleg Shekhtman, Georgios S. Sioutas, Pious D. Patel, Irina-Mihaela Matache, Mohamed M. Salem, Sonia Ajmera, Sandeep Kandregula, Jan-Karl Burkhardt, Visish M. Srinivasan

**Affiliations:** 1 Department of Neurosurgery, Perelman School of Medicine, University of Pennsylvania, Philadelphia, PA 19104, USA; 2 Department of Neurological Surgery, Thomas Jefferson University Hospital, Philadelphia, PA 19107, USA; 3 Department of Physiology, Faculty of Medicine, Carol Davila University of Medicine and Pharmacy, 050474 Bucharest, Romania

**Keywords:** aneurysm, vascular neurosurgery, endovascular, education, pipeline

## Abstract

Background: Aneurysm treatment options are rapidly evolving, as evidenced by the recent introduction and widespread adoption of flow diversion and intrasaccular devices. However, there is a need to understand how these newer technologies are used for difficult-to-treat aneurysms. The main aims of this study were to investigate the variation in aneurysm treatment recommendations among neurosurgeons, interventional radiologists, and interventional neurologists and to generally describe trends in endovascular treatment. Methods: In this survey-based study conducted from June to September 2024, participants were presented with clinical vignettes and asked to choose preferred treatment options, with responses analyzed based on demographic variables including specialty, age, and training prior to and after the introduction of flow diversion. Results: A total of 108 respondents completed the study with a representative mix of specialties—(45 (42.5%) radiologists, 22 (20.8%) neurologists, and 39 (36.8%) neurosurgeons. Sixty-six (61.1%) trained after the introduction of flow diversion. Treatment recommendations were significantly different by specialty (*p* < 0.001). The Kappa statistic to assess variation in responses showed significant variation in treatment preferences across aneurysm subtypes, ranging from poor (κ = 0.07) to fair (0.31). Treatment of ruptured aneurysms varied by specialty with radiologists opting for stent-assisted coiling at a higher rate than neurologists or neurosurgeons (*p* < 0.001). There was no significant difference in rates of recommending flow diversion or intrasaccular devices between those who had trained before and after their introduction (*p* = 0.97). Conclusion: The study highlights the dynamic nature of aneurysm management and considerable variability among different specialties. Further exploration into the rationale for each decision is needed to understand how specialty training affects these decisions.

## 1. Introduction

Approximately 3% of adults globally harbor an unruptured intracranial aneurysm. This prevalence can increase to 10% in certain high-risk groups [1]. From the introduction in 1995 of the detachable platinum coils, aneurysm management has undergone multiple paradigm shifts. Landmark randomized control trials demonstrated the safety and efficacy of coiling when compared to the gold standard of surgical clipping, ushering in the era of modern endovascular treatment [2]. Still, post-occlusion recanalization, wide-neck, and multi-lobed aneurysms remain a challenge and can represent around half of all ruptured saccular aneurysms. Today, the therapeutic arsenal includes various endovascular techniques in addition to coil embolization, including flow diverters, stent- and balloon-assisted coiling, and intrasaccular devices. These methods have been complemented by advancements in neuroimaging, allowing for more precise anatomical characterization and treatment planning [3,4].

In synergy with the shifting technological landscape, the demographic makeup of neuro-interventionalists has also evolved. While initially dominated by radiology faculty, in recent years, there has been an increased proportion of neurosurgeons and neurologists in endovascular training fellowships as faculty and fellows [5,6]. This confluence of specialties allows for a rich mixing of perspectives and expertise. Ostensibly, the significant differences in residency training inform long-term clinical decision-making.

Due to the rapid shifts, the selection of an appropriate therapeutic modality for aneurysm management remains a subject of debate and variability among practitioners [7,8,9]. This study aims to describe the variation in clinical decision-making among physicians and understand how the training period and specialty affect these decisions. We specifically focus on difficult-to-treat aneurysms, including wide-neck, multi-lobed, recurrent and/or large aneurysms, as we predict these will have the most variation.

## 2. Methods

### 2.1. Survey Study Creation

The survey consisted of nine basic demographic questions focusing on education and years in training, clinical experience, and current practice. To be included in this study, participants must be attending physicians who independently treat at least one aneurysm endovascularly a year. Participants who did not meet this criterion were excluded at the start of the survey. Following this, twenty clinical vignettes were presented, exploring preferred treatment options for each scenario. All cases were multiple-choice and exclusively featured endovascular options with the option to write in other modalities. Participants were required to analyze the vignettes based on brief patient data (age, sex), clinical history (diagnosis, aneurysm size, clinically important symptoms, rupture status, including days post-SAH and Hunt-Hess grade when applicable), as well as high-yield DSA and 3D DSA images. The patient dataset and images provided underwent back-testing by three dual-trained staff cerebrovascular neurosurgeons to confirm their sufficiency for making clinical decisions. The clinical cases were selected to encompass a broad range of aneurysm morphology, size, and shape. All respondent data were anonymized. The survey was accessible via Qualtrics (v3.2.0, Provo, UT, USA) and distributed via emails from an organizational database. The survey remained open from 1 June 2024 to 1 September 2024.

### 2.2. Analysis

Only responses that were greater than 50% complete were included to reduce non-response bias due to survey fatigue. Each response was treated as an independent observation. The respondents’ baseline characteristics were described using appropriate descriptive statistics.

Responses were all categorical variables and analyzed using X^2^ tests. The distribution of case answers was analyzed in a single variate model, looking at the effect of each of the demographic variables on the recommendation. Then cases with similar aneurysm morphology or location were grouped together, and the answers were analyzed in aggregate. Free-text responses with non-endovascular responses were not included in the analysis but are presented within descriptive tables. The Kappa statistic was used to determine agreement between respondents. Level of agreement followed the benchmark scale set by Landis and Koch, with 0.00–0.20 indicating poor, 0.21–0.40 fair, 0.41–0.60 moderate, 0.61–0.80 substantial, and 0.81–1.00 almost perfect agreement [10].

All statistical analyses and figures were performed with R Statistical Software (v4.1.2; R Core Team 2021).

## 3. Results

### 3.1. Basic Demographics

A total of 108 respondents were included in this study out of 5150 total possible respondents throughout the United States. 66 physicians (61.1%) had been in practice for 10 years or fewer. Three specialties were represented—45 (42.5%) radiologists, 22 (20.8%) neurologists, and 39 (36.8%) neurosurgeons. All specialists were attending physicians with training to independently perform endovascular aneurysm occlusion. Of the interventional neurologists, eleven reported treating 1 to 24 aneurysms, ten treating 25–100 aneurysms, and one treating more than 100 aneurysms a year. Of the interventional radiology respondents, nine reported treating 1 to 24 aneurysms, thirty reported 25–100 aneurysms, and six reported treating more than 100 aneurysms. For neurosurgeons, five reported treating 1 to 24 aneurysms, twenty-seven treated 25 to 100 aneurysms, and seven treated more than 100 aneurysms a year. Most physicians (69.4%) worked at centers with 50–200 endovascular aneurysms treated a year, and the majority (62.0%) personally treated 25–100 aneurysms a year. These baseline characteristics are summarized in Table 1.

### 3.2. Years in Practice

In aggregate, there was no significant difference in the distribution of answers when comparing physicians younger than 45 compared to older than 45 (*p* = 0.23). When comparing the proportion of respondents that would recommend coil embolization with or without stent-assisted coiling vs. flow diversion with a pipeline device or intrasaccular device, there was no significant difference between those in practice for ten years or fewer or greater than ten years (*p* = 0.97).

### 3.3. Ruptured Aneurysm Treatment

**Figure 1:**
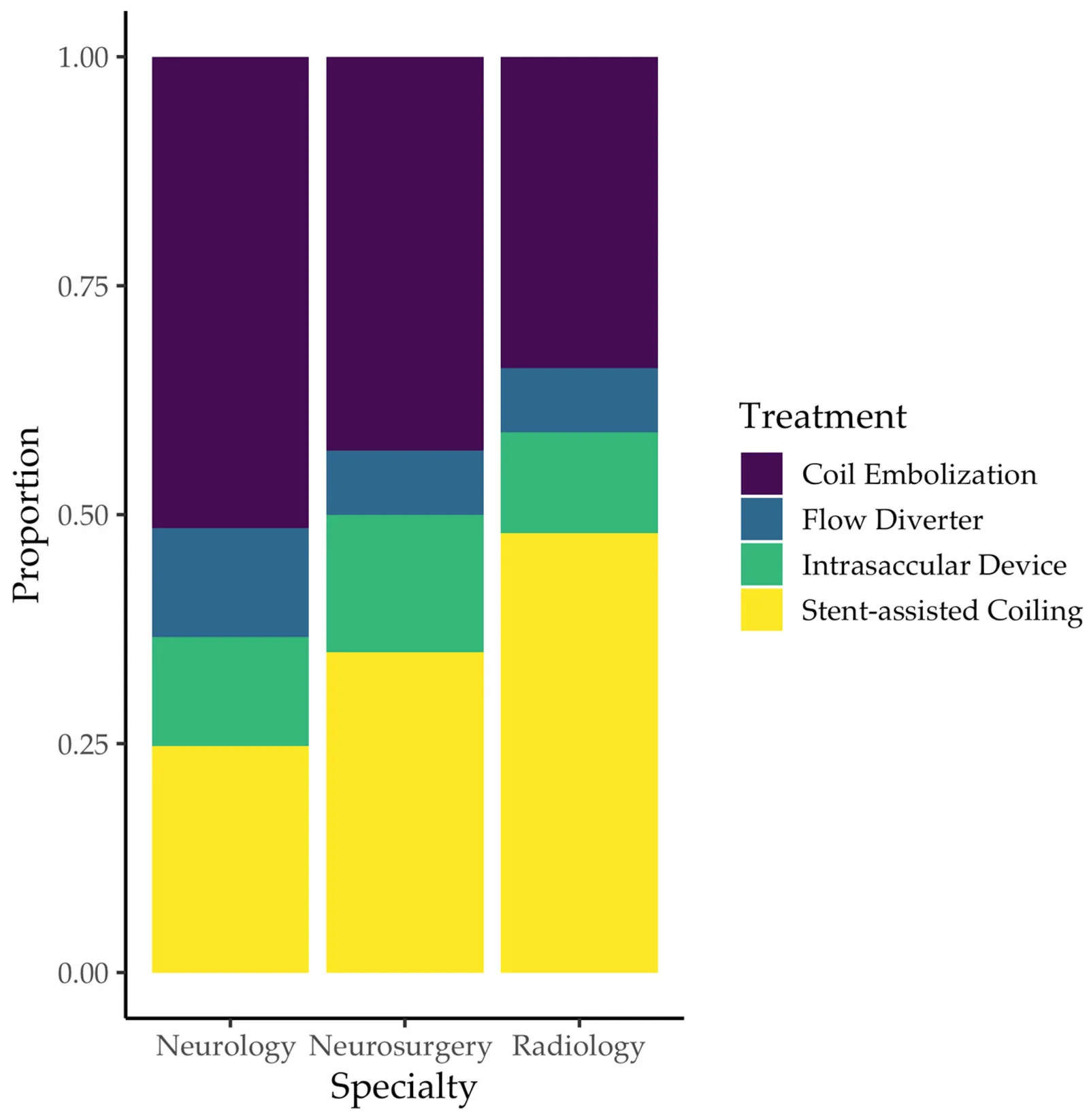
Proportion of treatment recommendations for ruptured aneurysms stratified by specialty.

Looking at aggregate answers to all ruptured aneurysm cases, 41.2% would coil embolize, 7.9% would place a flow diversion device, 12.3% would use an intrasaccular device, and 38.8% would use stent-assisted coiling. Overall agreement was poor (κ = 0.10). The proportion of answers was significantly different by specialty (*p* < 0.001), with a higher percentage of radiologists (47.8%) opting for stent-assisted coiling than neurosurgeons (35.1%) or neurologists (24.8%), and a higher percentage of neurologists opting for coil embolization (52.1%, neurosurgeons = (42.6%), radiologists (34.2%), Table 2, Figure 1. Specific locations and aneurysm types will be discussed in later sections.

### 3.4. Anterior Circulation Aneurysms

For a narrow-neck (neck/dome = 1.4), ruptured ACoA (anterior communicating artery) aneurysm without branching vessels, 60.4% of respondents would opt for coil embolization, 26.1% would use a stent-assisted coil, 12.5% would use an intrasaccular device and 1% would flow divert. For recurrence of this aneurysm, the majority (51.8%) would treat via stent-assisted coiling. For a wide-neck (neck = 5.25 mm), bilobed, ruptured ACoA aneurysm, the majority of radiologists (80.5%) and neurosurgeons (60.0%) would treat with stent-assisted coiling compared to only 38.9% of neurologists. A higher percentage of neurologists (44.4%) would coil embolize compared to 26.7.% of neurosurgeons and 7.3% of radiologists (*p* = 0.049). There were low levels of agreement between respondents regarding best treatment options for ruptured ACoA aneurysms (κ = 0.07).

For a narrow-neck, anterior choroidal artery (AChA) aneurysm with the AChA arising from aneurysm neck, 36% of respondents would use stent-assisted coiling, 32% coil embolization, and 32% flow diversion with no difference by specialty (*p* = 0.7) or age (*p* = 0.9). Responses differed by specialty for treatment options for a ruptured, narrow-neck, posterior communicating artery (PCoA) aneurysm. While most respondents would use coil embolization (neurosurgery = 61.3%, neurology = 57.9%, radiology = 50.0%), a higher proportion of neurologists (36.8%) would opt for flow diversion compared to neurosurgeons (22.6%) and radiologists (12.5%, *p* = 0.028).

There was no significant difference by specialty for a selection of unruptured, anterior circulation aneurysms. Overall agreement was higher for this subtype (κ = 0.31).

### 3.5. Posterior Circulation Aneurysms

**Figure 2:**
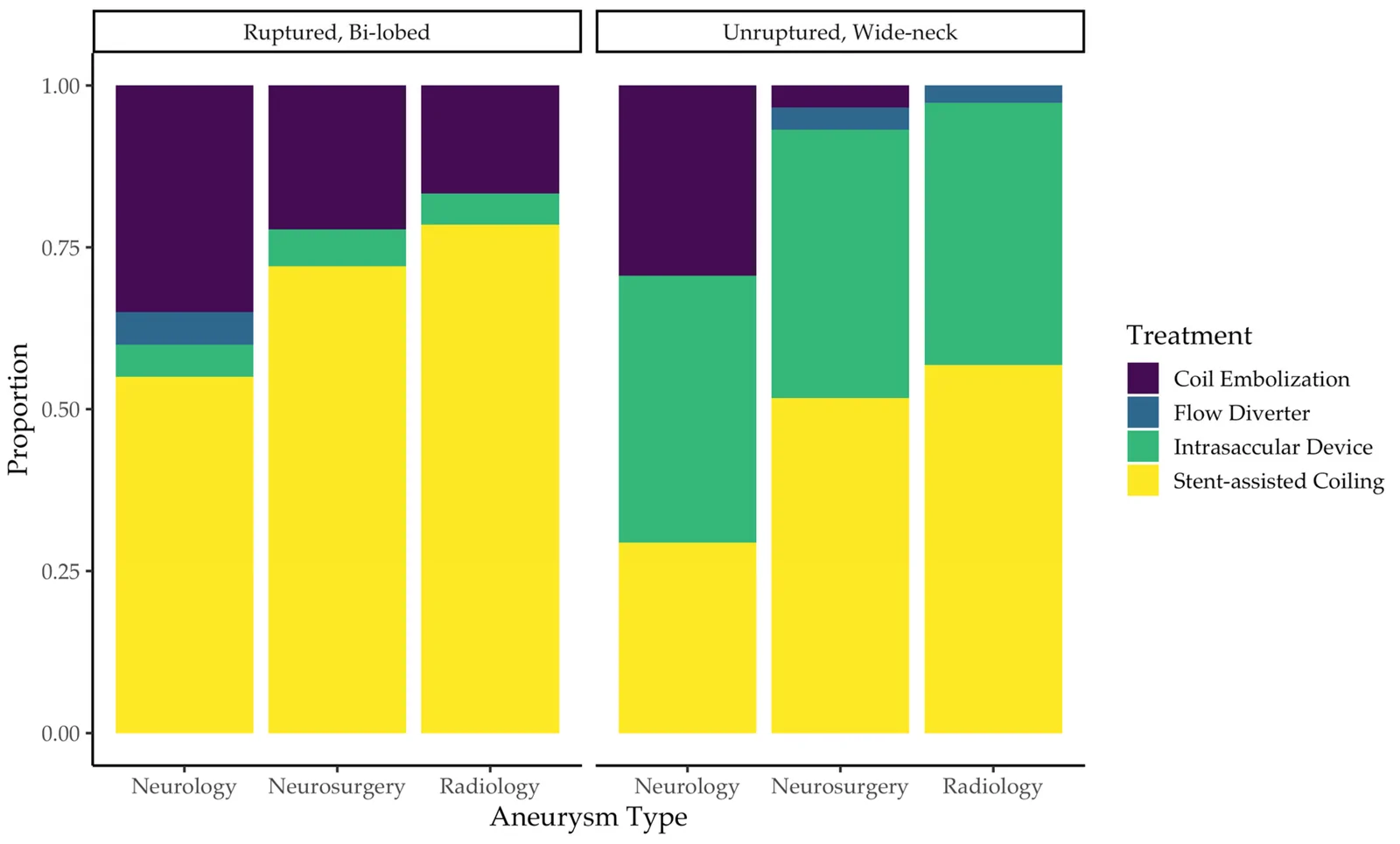
Proportion of treatment recommendations for two types of basilar tip aneurysms stratified by specialty.

For a ruptured, bi-lobed basilar tip aneurysm (8 × 6 × 7 mm), most respondents would use stent-assisted coiling (71.7%) vs. coil embolization (22.0%). There was a significant difference by specialty for treatment recommendations for a large, unruptured basilar tip aneurysm, with the majority of neurosurgeons (53.6%) and radiologists (58.3%) opting for stent-assisted coiling compared to only 29.4% of neurologists (*p* = 0.003). A higher proportion of neurologists would coil embolize (29.4%) compared to 3.6% of neurosurgeons and no radiologists. A comparable proportion of each specialty would use an intrasaccular device (neurology = 41.2%, neurosurgery = 42.9%, radiology = 41.7%). Responses are displayed in Figure 2.

Descriptive responses for these cases are summarized in Table 3.

### 3.6. Use of Intrasaccular Device

**Figure 3:**
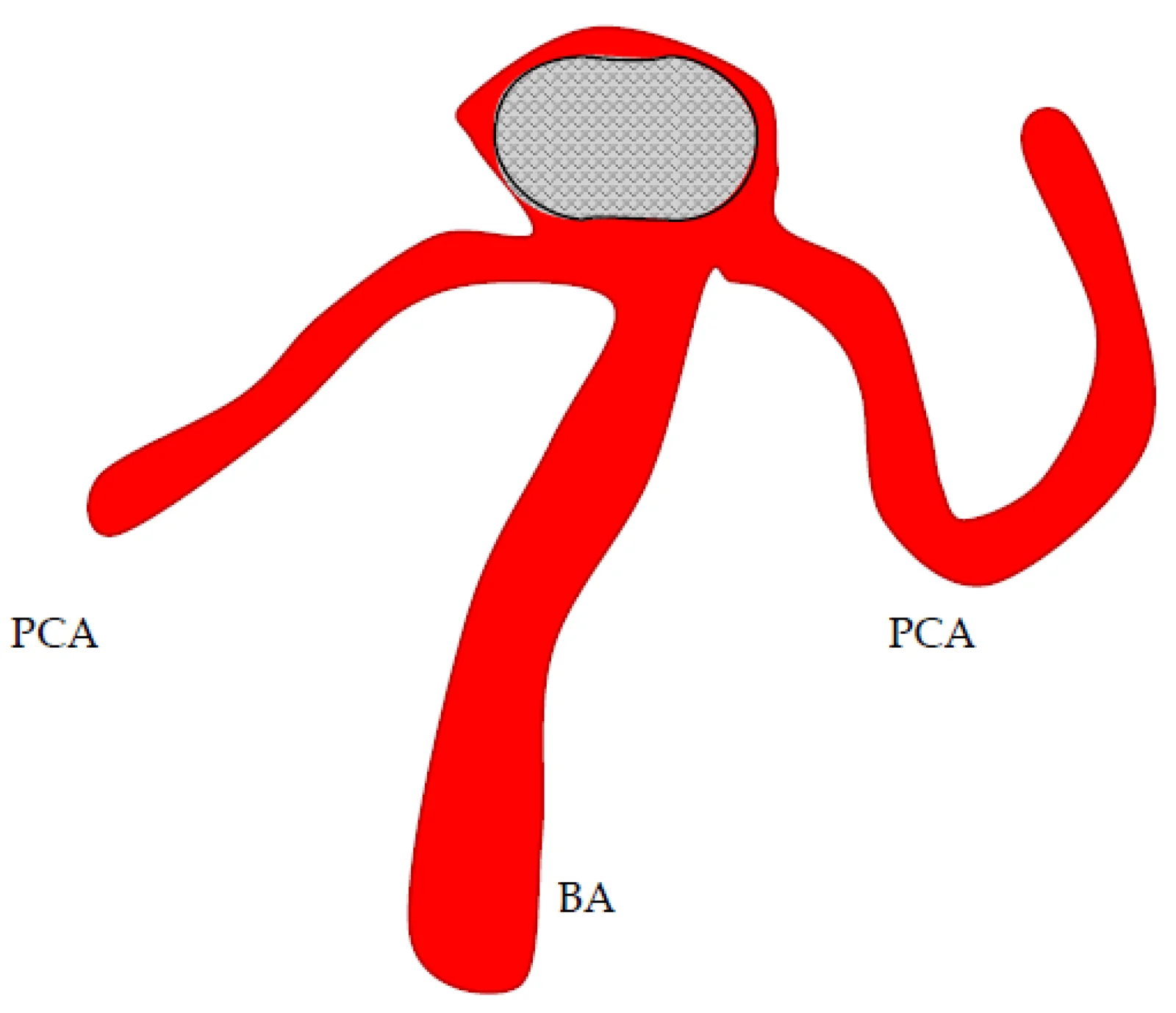
A general illustration of case number 9. PCA: Posterior Circulation Artery; BA: Basilar Artery.

Five aneurysm cases met Food and Drug Administration (FDA) criteria for intrasaccular device treatment. When analyzing answers for the 5 cases in aggregate, more than 10% of respondents would place an intrasaccular device. Twelve respondents (11.3%) would place an intrasaccular device for an unruptured anterior communicating artery aneurysm, 36 (36.7%) for a ruptured middle cerebral artery bifurcation aneurysm, 11 (11.7%) for a ruptured anterior communicating artery aneurysm, 36 (39.6%) for an unruptured giant basilar tip aneurysm, and 18 (19.8%) for a ruptured internal cerebral artery aneurysm. In aggregate, 88% of respondents would either coil embolize (with or without stent assistance) or place an intrasaccular device for these aneurysm types. Within those responses, there was no significant difference by specialty (*p* = 0.146) for treatment choice. Figure 3 shows a general illustration of case number 9 (wide-neck, unruptured basilar tip aneurysm) and how an intrasaccular device could be deployed to treat this aneurysm.

## 4. Discussion

This is the first study to illustrate variation in specific endovascular treatment recommendations for ruptured and unruptured aneurysms. Previous studies have looked more generally at differentiating surgical vs. endovascular treatment or the use of different types of flow diversion devices [8,9,11,12]. Due to the frequent paradigm shifts in aneurysm occlusion options, we wanted to know how training prior to the introduction of flow diversion and intrasaccular devices affects treatment recommendations [13,14]. For this study, we defined those who trained in the pre-flow diverter era as those who had completed training ten years ago. We did not find significant differences in treatment choice between these two populations, even when stratifying for specialty. Likely interventionalists are a self-selecting group of people who are early adopters of new technology, with the comfort and desire to incorporate these into their practice. Training at a time when these devices were not yet in use did not deter them from considering these techniques for aneurysm occlusion. This dynamism is reflected throughout the field as new devices and techniques are rapidly tested and adopted [15,16].

The treatment of ruptured aneurysms remains a controversial topic. Stent-assisted coiling and flow diverters are generally avoided in acute rupture patients due to the necessity of antiplatelet therapy. Antiplatelet therapy puts patients at increased risk of hemorrhagic complications secondary to infarction from vasospasm, or the need that these critically ill patients will need a variety of emergent procedures, including ventriculostomy, craniotomy, and/or central venous access [17]. Interestingly, we did see a significant difference by specialty, with a higher proportion of radiologists opting for stent-assisted coiling compared to neurologists and neurosurgeons. This difference may reflect a difference in the level of involvement in post-procedural care and in the management of complications. A recent propensity-matched analysis showed comparable and low hemorrhagic and thromboembolic complications after stent-assisted coiling vs. coiling alone for ruptured aneurysms [18]. This is another area that needs further inquiry to understand how interventionalists weight the risks and benefits of stent-assisted coiling with dual antiplatelet treatment and larger prospective trials to understand the true risks of stent-assisted coiling.

Understanding the role of endovascular treatment options for ACoA aneurysms is important, as these account for 50% of aneurysm ruptures. While larger studies have focused on coiling vs. microsurgical management of these aneurysms, the recent literature has shown an expanding role for the usage of stent-assisted coiling, flow diversion, and intrasaccular devices [19,20,21,22,23,24]. However, the efficacy of these newer techniques compared to traditional coiling or surgical treatment is unknown. The significant variation in responses reflects this uncertainty about the best treatment modality for each of these ACoA subtypes and warrants further investigation.

Both surgical and endovascular treatment of anterior choroidal artery aneurysms can have significant complications related to compromising choroidal circulation [25]. Aneurysms arising from the AChA branch (Type 2) vs. the ICA adjacent to the AChA take off (Type 1) are at higher risk of post-operative stroke [26]. Symptomatic ischemic complications from treatment of this anatomical subtype can be as high as 16% with surgical treatment [27] and 7% with endovascular coiling [26]. We wanted to know treatment preferences for a Type 2 (higher risk), ruptured AChA aneurysm. While the majority would use coil embolization or stent-assisted coiling, 1/3 of our respondents would use flow diversion for a ruptured, Type 2 aneurysm. Recent studies looking at unruptured AChA aneurysms demonstrate good occlusion and safety profiles with flow diversion [28,29]. No study has yet shown the safety and efficacy of flow diversion for a Type 2, ruptured AChA aneurysm. Though responses here are hypothetical, the large proportion that would choose this modality over other methods with previously documented efficacy shows a need for further evaluation of an expanded role for flow diversion.

We found a preference for stent-assisted coiling for treatment of basilar tip aneurysms, though there was variation by specialty. Notably, this is in line with a large, retrospective series showing lower recurrence, retreatment, and re-hemorrhage rates for basilar tip aneurysms treated with stent-assisted vs. non-stent-assisted coiling [30]. There have been few published cases showing the efficacy of an intrasaccular device for the treatment of basilar tip aneurysms. 41.4% of our respondents would advocate for their use in a wide-neck basilar tip aneurysm, highlighting another area meriting further study [31].

For the treatment of wide-neck aneurysms, intrasaccular devices offer the additional benefit of avoiding potential risk from dual antiplatelet therapy (DAPT). These devices are self-expanding constructs that disrupt incoming blood flow into an aneurysm. By disrupting the inflow, an organized clot forms inside the aneurysm sac until it becomes occluded. Since the device sits inside the aneurysm sac and does not enter the parent vessel, no DAPT treatment is needed after [32]. Figure 3 is an illustration of how an intrasaccular device may be used for a basilar terminus aneurysm. The figure shows the basilar artery bifurcating into the posterior cerebral arteries with a large, wide-necked aneurysm at the basilar terminus. The intrasaccular device fills the aneurysm cavity and provides flow-disruption across the aneurysm neck. Currently, the most widely used intrasaccular device, woven endo-bridge (WEB), is approved for the treatment of wide-neck bifurcation aneurysms that occur at the internal carotid artery terminus, middle cerebral artery bifurcation, anterior communicating artery complex, or basilar artery apex [33]. Our responses show a notable proportion of physicians would place an intrasaccular device for each of the above indications, with the greatest proportion recommending intrasaccular devices for MCA bifurcation and unruptured giant basilar tip aneurysms. Interestingly, there was no significant difference between the usage of WEB devices between physicians who were trained before and after the introduction of these devices. Recent studies show an expanding role for these devices, and our study shows that many already recommend it for off-label indications over standard treatment modalities [34,35,36,37]. Further work is needed to understand the full scope of off-label.

The wide variety of physician demographics is a key strength of this study that improves its generalizability. The proportion of respondents from each specialty (42% radiology, 37% neurosurgery, and 21% neurology) was similar to that of prior studies analyzing the demographics of neuro-interventionalists in the USA and Canada, suggesting a representative sample by specialty breakdown [38,39]. Physicians also had a wide range of years in practice and age, which was key to studying how practices evolved in a rapidly changing field.

Limitations of this study include response bias in this voluntary survey, with an overall low response rate. Participants self-identified, so no information is available about non-responders. Therefore, this study provides preliminary information on preferences to guide future studies rather than generalizable conclusions. Given the original aim to focus on endovascular options for treatment, surgical treatment was not a default option but was able to be answered within the umbrella of “other treatment.” Therefore, no conclusions can be drawn on endovascular vs. surgical treatment and as such our results focused on different endovascular options rather than the choice of endovascular vs. surgical. The answers were self-reported rather than a survey of actual clinical practice. To maintain the anonymity of participants, no information on true personal or center volume is available. Additionally, due to time constraints with a long survey, truncated clinical information was provided. It is possible that physicians answered based on best practices rather than their true clinical practice. Patients with subarachnoid hemorrhage from a ruptured aneurysm are clinically complicated and can present in a variety of clinical conditions with multiple comorbidities. This survey study captured decision-making based on a truncated vignette, and it is possible that other clinical variables will change decision-making in true clinical practice.

## 5. Conclusions

This study shows preliminary evidence that there is significant variation in reported preferences for endovascular aneurysm occlusion. Some of this variation occurs between specialty types, but overall, there is a wide range of preferences when considering difficult-to-treat aneurysms. The findings underscore the dynamic nature of the field, where interventionalists demonstrate a willingness to incorporate new technologies into their practice regardless of when they received their training. Moreover, the study highlights ongoing debates surrounding the management of ruptured aneurysms. Importantly, respondents showed preferences for endovascular treatment modalities for clinical situations not yet studied in the literature, highlighting several areas meriting further investigation. Further studies are needed to see true variations in trends in clinical practice.

## Figures and Tables

**Table 1:** Basic demographic information of survey respondents.

Total Respondents	*n* = 108
Age (years)	
Greater than 61	21 (19.4)
45 to 60	28 (25.9)
Less than 45	59 (54.6)
Years in Practice	
10 or more	42 (38.9)
Less than 10	66 (61.1)
Specialty	
Neurology	22 (20.8)
Neurosurgery	39 (36.8)
Radiology	45 (42.5)
Personal Volume (aneurysms treated/year)	
More than 100	15 (13.9)
25–100	67 (62.0)
Less than 25	25 (23.1)
Center Volume (aneurysms treated/year)	
more than 200	23 (21.3)
50–200	75 (69.4)
less than 50	10 (9.3)

**Table 2:** Top four treatment recommendations for ruptured aneurysm occlusion stratified by specialty.

Treatment (*n*, %)	Neurology (*n* = 165)	Neurosurgery (*n* = 242)	Radiology (*n* = 368)	*p*-Value
Coil Embolization	86 (52.1)	103 (42.6)	126 (34.2)	<0.001
Flow Diverter	19 (11.5)	18 (7.4)	25 (6.8)	
Intrasaccular Device	19 (11.5)	36 (14.9)	41 (11.1)	
Stent-assisted Coiling	41 (24.8)	85 (35.1)	176 (47.8)	

**Table 3:** Descriptive information about treatment choices for selected cases. (ACoA = Anterior Communicating Artery, AChA = Anterior Choroidal Artery, PCoA = Posterior Communicating Artery, ICA = Internal Carotid Artery, M1 = Middle Cerebral Artery, Segment 1, BA = Basilar Artery).

	Treatment Choice, *n* (%)						
Aneurysm Description	Coil Embolization	Stent-Assisted Coiling	Flow Diverter	Intrasaccular Device	Non-Operative	Micro-Surgical	Total
1. Narrow-neck, ruptured ACoA (3.5 × 2.5 mm)	58 (54.7)	27 (25.5)	1 (0.9)	12 (11.3)	1 (0.9)	7 (6.6)	106
2. Remnant neck after treatment of Case 1	19 (19.8)	45 (46.9)	19 (19.8)	4 (4.2)	0 (0.0)	9 (9.4)	96
3. Wide-neck, bilobed, ruptured ACoA (2 × 3 mm)	19 (19.0)	59 (59.0)	4 (4.0)	8 (8.0)	1 (1.0)	9 (9.0)	100
4. Narrow-neck, ruptured AChA (Type 2)	25 (24.0)	27 (26.0)	24 (23.1)	2 (1.9)	7 (6.7)	19 (18.3)	104
5. Narrow-neck, ruptured PCoA (5.5 × 2.2 mm)	51 (49.0)	21 (20.2)	19 (18.3)	4 (3.8)	1 (1.0)	8 (7.7)	104
6. Narrow-neck, unruptured ophthalmic ICA (4 × 2 mm)	3 (2.9)	11 (10.5)	45 (42.9)	1 (1.0)	44 (41.9)	1 (1.0)	105
7. Narrow-neck, unruptured M1 (3 × 2 mm)	8 (8.7)	4 (4.3)	30 (32.6)	1 (1.1)	41 (44.6)	8 (8.7)	92
8. Wide-neck, bilobed, ruptured BA tip (8 × 6 × 7 mm)	22 (21.8)	71 (70.3)	1 (1.0)	5 (5.0)	0 (0.0)	2 (2.0)	101
9. Wide-neck, unruptured BA tip (10 × 11 × 9 mm)	6 (6.9)	41 (47.1)	2 (2.3)	36 (41.4)	1 (1.1)	1 (1.1)	87

## Data Availability

Data available upon reasonable request from the corresponding author.
